# A Possible Association Between Zika Virus Infection and CDK5RAP2 Mutation

**DOI:** 10.3389/fgene.2021.530028

**Published:** 2021-03-19

**Authors:** Estephania Candelo, Ana Maria Sanz, Diana Ramirez-Montaño, Lorena Diaz-Ordoñez, Ana Maria Granados, Fernando Rosso, Julian Nevado, Pablo Lapunzina, Harry Pachajoa

**Affiliations:** ^1^Universidad Icesi, Ear Institute University College London and Fundación Valle del Lili, Cali, Colombia; ^2^Center for Research on Congenital Anomalies and Rare Diseases (CIACER), Department of Basic Medical Sciences, Universidad Icesi, Cali, Colombia; ^3^Fundacion Valle del Lili, Cali, Colombia; ^4^Neuroradiology Department Fundación Valle del Lili, Cali, Colombia; ^5^Instituto de Genética Médica y Molecular (INGEMM), IdiPAZ, HospitalUniversitario La Paz, Madrid, CIBER de Enfermedades Raras (CIBERER), ISCIII, Madrid, Spain; ^6^Genetics Department, Fundación Valledel Lili, Cali, Colombia

**Keywords:** Colombia, microcephaly, whole-exome sequencing, Zika virus, vertical transmission, brain abnormalities, CDK5RAP2

## Abstract

**Introduction:**

Flaviviridae family belongs to the Spondweni serocomplex, which is mainly transmitted by vectors from the *Aedes* genus. Zika virus (ZIKV) is part of this genus. It was initially reported in Brazil in December 2014 as an unknown acute generalized exanthematous disease and was subsequently identified as ZIKV infection. ZIKV became widespread all over Brazil and was linked with potential cases of microcephaly.

**Case report:**

We report a case of a 28-year-old Colombian woman, who came to the Obstetric Department with an assumed conglomerate of fetal abnormalities detected *via* ultrasonography, which was performed at 29.5 weeks of gestation. The patient presented with multiple abnormalities, which range from a suggested Arnold–Chiari malformation, compromising the lateral and third ventricles, liver calcifications, bilateral pyelocalic dilatations, other brain anomalies, and microcephaly. At 12 weeks of gestation, the vertical transmission of ZIKV was suspected. At 38.6 weeks of gestation, the newborn was delivered, with the weight in the 10th percentile (3,180 g), height in the 10th percentile (48 cm), and cephalic circumference under the 2nd percentile (31 cm). Due to the physical findings, brain magnetic resonance imaging (MRI) was performed, revealing a small and deviated brain stem, narrowing of the posterior fossa, a giant posterior fossa cyst with ventricular dilatation, a severe cortical and white matter thinning, cerebellar vermis with hypoplasia, and superior and lateral displacement of the cerebellum. In addition, hydrocephalus was displayed by the axial sequence, and the cerebral cortex was also compromised with lissencephaly. Schizencephaly was found with left frontal open-lip, and no intracranial calcifications were found. Two novel heterozygous nonsense mutations were identified using whole-exome sequencing, and both are located in exon 8 under the affection of ZIKV congenital syndrome (CZS) that produced a premature stop codon resulting in the truncation of the cyclin-dependent kinase 5 regulatory subunit-associated protein 2 (CDK5RAP2) protein.

**Conclusion:**

We used molecular and microbiological assessments to report the initial case of vertically transmitted ZIKV infection with congenital syndrome associated with a neurological syndrome, where a mutation in the *CDK5RAP2* gene was also identified. The *CDK5RAP2* gene encodes a pericentriolar protein that intervenes in microtubule nucleation and centriole attachment. Diallelic mutation has previously been associated with primary microcephaly.

## Introduction

Flavivirus commonly spreads through vectors from the *Aedes* genus (Hayes, 2009). Zika virus (ZIKV) is a member of this family. It was first reported in Brazil in December 2014 as an unknown generalized exanthematous disease and was later described as ZIKV infection (Lowe et al., 2018). By May 2015, ZIKV had become widespread in Brazil and was linked with potential microcephaly cases in November 2015. Subsequently, ZIKV disseminated rapidly to other South American and Caribbean countries in the majority of the territory and across 22 territories around Brazil, where the vector was already present. By January 2016, almost 30,000 cases of ZIKV infection had been revealed (Candelo et al., 2019). After the Americas outbreak, the WHO declared a public health emergency of international interest in February 2016 (World Health Organization [WHO], 2016).

ZIKV infection was declared a public health emergency when strong evidence was associated with the hypothesis that it caused microcephaly and congenital abnormalities during pregnancy and increasing evidence supported this link with the detection of ZIKV in fetal brain tissue (Mlakar et al., 2016; Schuler-Faccini et al., 2016; Ventura et al., 2016; Wang and Ling, 2016; Passi et al., 2017), amniotic fluid (Calvet et al., 2016), and placenta, which also supported the vertical transmission of ZIKV (Besnard et al., 2014). Further research using *in vitro* and *in vivo* studies has established that the virus is highly neurotropic (Garcez et al., 2016; Sirohi et al., 2016; Tang et al., 2016; Ahmad et al., 2017). A recent study shows that ZIKV targets neuroprogenitor cells, which are derived from pluripotent stem cells, from which the ZIKV particles are released. When neuroprogenitor cells are infected by the ZIKV particles, the apoptotic process is activated, and it intensifies cell death and disrupts the cell cycle resulting in decreased neuroprogenitor cell growth, most likely due to transcriptional dysregulation in cell cycle-related pathways (Tang et al., 2016).

Before 2015, the yearly number of cases of microcephaly in Brazil was repeatedly less than 200 (Cosme et al., 2017). Furthermore, 4,783 suspected cases of microcephaly were reported between November 2015 and January 30, 2016, including newborn and fetal losses. From these cases, only 36.6% (404) were classified as confirmed cases of microcephaly from the 1,103 cases that had undergone clinical, laboratory, and imaging examinations. ZIKV was only detected in 15 newborn and 2 fetal losses (1.54%). The majority of the probable cases were under investigation, and a considerable proportion represented misdiagnosis and overreporting noise due to uncertainty, with both variables probably inflating the prevalence (Victora et al., 2016). In humans, classic Zika fever is a self-limiting sickness characterized by fever, maculopapular rash, headache, conjunctivitis, and myalgia (Ioos et al., 2014). Clinical manifestation only occurs in 20% of the population affected, and the majority of infections are asymptomatic. A serological survey conducted in Salvador in Northeast Brazil indicated a peak seroprevalence of 63% in 2016, with 205,578 and 13,353 cases of ZIKV infection reported in 2016 and 2017, respectively (Netto et al., 2017; Secretaria de Vigilância em Saúde-Ministério da Saúde, 2017).

The ZIKV epidemic in Colombia started in August 2015, but laboratory evidence of ZIKV infection was not reported until October 2015. By April 2016, 65,726 suspected cases of ZIKV had been reported, of which only 2,485 (4%) were confirmed by reverse-transcription (RT)-polymerase chain reaction (PCR) assay. Furthermore, 11,944 pregnant women were reported as potential cases of ZIKV infection, but only 1,484 (12%) of these were confirmed. From this population, 50 newborns were reported as possible ZIKV congenital syndrome (CZS), but ZIKV infection was confirmed in only 4 (8%) of them (Pacheco et al., 2016).

In this case report, we used molecular, microbiological, and genetic assessments to characterize the first known case of vertically transmitted ZIKV infection and congenital syndrome associated with a neurological syndrome, where a mutation in cyclin-dependent kinase 5 regulatory subunit-associated protein 2 (CDK5RAP2) was identified. The *CDK5RAP2* gene encodes a pericentriolar protein that mediates microtubule nucleation and centriole attachment (Fong et al., 2008), and diallelic variants of this gene have been priorly linked with isolated microcephaly syndrome.

## Case Report

In mid-2016, a 28-year-old previously healthy Colombian woman with later prenatal care attention came to the Perinatology and Obstetric Department at Fundación Valle del Lili-University Hospital due to numerous fetal abnormalities observed in ultrasonography done at 29.5 weeks of gestation, which demonstrated brain anomalies with a possible Arnold–Chiari malformation, compromised lateral and third ventricles, liver calcifications, bilateral pyelocaliceal dilatations, microcephaly, and an apparent clubfoot. All relevant antecedents during pregnancy were considered (smoking, alcohol, drugs, or any other perinatal infection). The patient had experienced an episode of illness for 2–7 days that was characterized by fever, myalgias, and vomiting. She went to the hospital, where ZIKV was presumed, and RT-PCR of the amniotic fluid was done, with a positive result at 12 weeks of gestation. The findings confirmed ZIKV vertical transmission and congenital infection. Inadequate prenatal follow-ups were performed until the fetal examination at 29.5 weeks, which showed multiple malformations.

At the time of delivery, the mother was traveling from a remote area of the country. She went into labor on the way to the hospital and gave birth in the ambulance. According to the medical records and first ultrasonography date, delivery was at 38.6 weeks of gestation. The male newborn weighed 3,180 g (10th percentile), with a height of 48 cm (10th percentile) and a cephalic circumference of 31 cm (under the 2nd percentile) according to the WHO Growth Charts from the Centers for Disease Control and Prevention (CDC) (WHO Child Growth Standards, 2019; World Health Organization, 2019). Transfontanelle ultrasonography reported microcephaly, an increase of the width and number of the circumvolutions due to pachygyria, and ventriculomegaly in the infratemporal and supratemporal fossae. There was also a non-discharging cerebrospinal fluid-filled cleft lined with gray matter on the left lateral ventricle going to the cerebral cortex, closed-lip schizencephaly, subcortical echogenic and punctiform, cystic image on the posterior fossae communicated with the fourth ventricle, thin corpus callosum, and pathological calcification in the liver, brain (cortical and subcortical), and placenta. As part of the evaluation approach to a newborn with brain abnormalities, TORCH testing was performed to rule out the most frequent congenital infections (syphilis, toxoplasmosis, rubella, hepatitis B, HIV, cytomegalovirus, herpesvirus 1 and 2, and parvovirus B19) as causal etiology.

**FIGURE 1:**
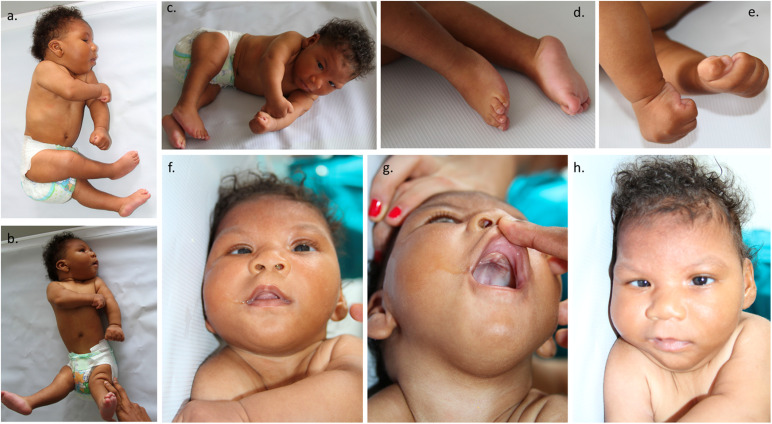
Representation of the patient phenotype. **(a,b)** The patient showed distal tremors, hypertonia, trunk hyperextension, spasticity, microcephaly, craniofacial disproportion, and a decreased vertical skull size. **(c)** Multiple dimples and arthrogryposis. **(d)** Feet contractures and prominent calcaneus. **(e)** Clenched fists, hand contractures, and camptodactyly. **(f)** Strabismus. **(g)** Excess skin over the entire scalp and forehead and occipital and nuchal skin folds, generating the appearance of a short neck. **(h)** Sloping of the forehead and prominence of supraorbital ridges, which creates an appearance of proptosis and oversized facial features, epicanthal folds, and retrognathia. Bilateral depressions were present on the forehead.

The mother reported that the newborn was irritable, with an abnormal pattern of crying and hyperexcitability. The physical examination showed distal tremors, hypertonia, trunk hyperextension, spasticity, increased deep tendon reflexes, persistent primitive responses, clenched fists, strabismus, and nystagmus. Microcephaly was present, which was characterized by craniofacial disproportion, decreased vertical size of the skull, a sloping forehead, and pronounced supraorbital ridges, giving the appearance of oversized facial features and proptosis. Epicanthal folds, retrognathia, excess skin over the entire scalp and forehead, and occipital and nuchal skin folds were also found, giving the appearance of a short neck. The forehead presented with bilateral depressions and prominent overlapping metopic and sagittal sutures with supratemporal depressions and a large visible occipital prominence. An examination of the extremities revealed hand contractures, camptodactyly, feet contractures, prominent calcanei, multiple dimples, and arthrogryposis (Figure 1). Because of the multiple abnormalities, a complete genetic assessment was performed on the newborn and his mother.

**FIGURE 2:**
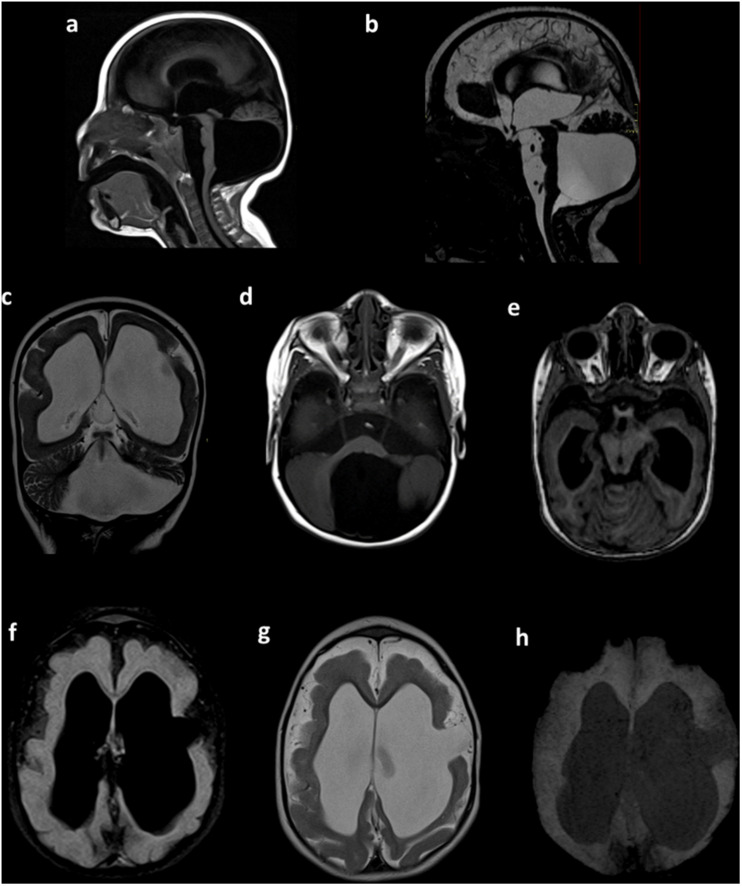
Brain MRI. Sagittal T1-weighted **(a)** and sagittal constructive interference in steady state (CISS) **(b)** sequences showing microcephaly with a giant posterior fossa cyst, a small and displacement of the brain stem, and tightening of the posterior fossa. Coronal T2-weighted images **(c)** showing ventricular dilatation, cortical and white matter narrowing, and posterior fossa cyst. Axial T1-weighted images **(d)** showing a posterior fossa cyst. Axial T1-weighted multiplanar reconstruction **(e)** showing a narrow posterior fossa, cerebellar vermis with hypoplasia, and superior and lateral deviation of the cerebellum. Axial FLAIR **(f)** and axial T2-weighted sequences **(g)** showing hydrocephalus and anomalous cerebral cortex with lissencephaly and left frontal open-lip schizencephaly. Axial susceptibility-weighted image **(h)**, with the absence of intracranial calcifications. Preliminary data of this study were previously presented in the IBRO meeting 2019 (Candelo et al., 2019).

After 1 year of follow-up, a complete checkup was performed. The findings showed irritability, an abnormal pattern of crying, hyperexcitability, hypertonia, spasticity, clenched fists, strabismus, clinodactyly of the fifth finger, and an ogival palate. Microcephaly was still present, with a few of the previously observed characteristics, such as a small skull, retrognathia, and a prominent metopic suture. A physical examination of the extremities found hand contracture and camptodactyly, feet contracture, prominent calcanei, multiple dimples, arthrogryposis, and a severe delay in the developmental milestones. A brain magnetic resonance imaging (MRI) showed microcephaly, with a giant posterior fossa cyst, ventricular dilatation, hypoplasia of the cerebral vermis, and an anomalous cerebral cortex with lissencephaly (Figure 2).

## Methods

### ZIKV Sample Collection

Following the National Institutes of Health recommendations, amniocentesis was performed at 12 weeks of gestation for ZIKV infection case suspicions. Ultrasound-guided transabdominal amniocentesis was done, and around 5 ml of amniotic liquid was aspirated and instantly stored at −80°C for testing. Further samples were taken from the cerebrospinal fluid when the infant was born, and the blood, amniotic fluid, and placenta were also taken, as is previously described in Candelo et al. (2019).

### ZIKV Detection

The molecular identification of ZIKV in the amniotic fluid and cerebrospinal fluid was done by using a SuperScript III Platinum One-Step RT-PCR system (Invitrogen, Carlsbad, CA, United States) run by the Colombian Government National Institutes of Health using the primers ZIKV 1087, ZIKV 1163, and ZIKV 1108-FAM, followed by the Lanciotti protocol (Lanciotti et al., 2008). For hybridization and extension, an ABI 7500 Real-Time PCR System (Thermo Fisher Scientific, Waltham, MA, United States) was operated (Pacheco et al., 2016).

Samples were obtained from the mother and the newborn and those from viral isolation trials for ZIKV RNA, using a TaqMan RT-PCR assay and quantitative RT-PCR for ZIKV following the CDC protocol (Centers for Disease Control and Prevention, 2019). Standard methods were used to determine the levels of ZIKV IgM, IgG, and neutralizing antibody titers. Additionally, we tested for other flaviviruses and arthropod-borne viruses. In addition, the antibody levels of infectious diseases that might produce congenital abnormalities, such as toxoplasma, cytomegalovirus, syphilis, herpes, rubella, hepatitis, parvovirus B19, and HIV, were tested in the mother and the newborn (TORCH test), as is described previously in the literature (Candelo et al., 2019).

### Karyotyping and Array Comparative Genomic Hybridization Analysis

The classical cytogenetics G-banding technique was performed after the patient had given birth to do a blood karyotype analysis. The karyotype analysis was performed on cells in metaphase using a microscope and the CytoVision (Leica) karyotype software system. After extracting DNA from the patient’s peripheral blood cells, 1 μg was used for array comparative genomic hybridization (a-CGH) utilizing KaryoArray 3.0 (8 × 60K; Agilent Technologies, Santa Clara, CA, United States) and was marked by fluorescence to compare the patient’s DNA with the control sample. DNA hybridization was done with a human genomic microarray of 860 K oligonucleotides using commercially available Agilent-based arrays, which were analyzed using an Agilent scanner with Feature Extraction 9.1 software. The aberration detection method 2 algorithm was used to determine any statistically significant aberrations. This was defined as the minimum number of oligonucleotides to consider an alteration. Subsequently, the medium resolution of the array was one oligonucleotide per 9 Kb in the regions of maximum interest, such as microdeletions and microduplications, centromeres, and telomeres. The algorithm in CGH Analytics version 3.5 software (Agilent Technologies) was used (Blanco-Kelly et al., 2017), as is described previously in the literature (Candelo et al., 2019).

### Whole-Exome Sequencing Methods

The whole-exome sequencing (WES) was captured by using a SureSelect Human All Exon capture kit (Agilent) of 51 Mb, and sequencing was done by using an Illumina Hiseq 2000 sequencing system (Illumina, San Diego, CA, United States). During the sequencing, paired readings of 101 nucleotides in length were acquired. The different variants were analyzed by focusing on genes related to microcephaly. Only variants in the coding region and flanking intronic regions with minor allele frequency <1% were evaluated and compared using datasets acquired from the 1000 Genomes Project Consortium, dbSNP, Exome Variant Server, and Exome Aggregation Consortium databases. Variants in around 20 bp of flanking intron regions were examined and sequenced by Sanger sequencing (Candelo et al., 2019). Parental carrier status of the clinical relevant variants was confirmed by Sanger Sequencing analysis, as is described previously in the literature (Candelo et al., 2019).

### *In silico* Analysis

Two heterozygous nonsense mutations in CDK5RAP2 were analyzed with variant functional prediction software tools (DANN, MutationTaster, Condel, SIDT, and FATHMM) in order to predict the pathogenicity of the mutations. In addition, these variants were searched in Human Gene Mutation Database (HGMD), Leiden Open Variation Database (LOVD), and ClinVar database, as is described previously in the literature (Candelo et al., 2019).

### Tissue Expression of CDK5RAP2

Isolation of total RNA from the patient’s and parents’ mouth swab was done with the TRIzol reagent (Life Technologies, CA, United States). RNA integrity and concentrations were both analyzed using a 1% agarose gel and Nanodrop^®^ ND-1000 Spectrophotometer (Thermo Fisher Scientific, MA, United States). RNA reverse transcription was performed by a High-Capacity cDNA Reverse Transcription Kit (Applied Biosystems, CA, United States) using 100 ng of total RNA according to the manufacturer’s instructions. For quantitative PCR (qPCR), cDNA was amplified using CDK5RAP2 primers 5′-GTTGGGGAAATGGTCTGCTCC-3′ and 3′-TATGTTCAGTGGGGCCATGA-5′. PCR amplification was performed in a 20-μl volume that contained: 10 μl of the EXPRESS SYBR^®^ GreenER^TM^ qPCR SuperMixes (0.25×) kit (Applied Biosystems, CA, United States), 0.4 μl of each of the primers (1×), 0.04 μl ROX (50 nM), 1 μl of cDNA, and 8.16 μl of water. Real-time PCR was performed in a 7500-Fast real time PCR instrument, and reaction was carried out with the following conditions: 95°C for 20 s, followed by 40 cycles of: 95°C for 1 s and 60°C for 20 s and for the melt curve: 95°C for 15 s, 60°C for 60 s, 95°C for 15 s, and 60°C for 15 s. GAPDH mRNA was used as an internal control (house-keeping gene), and the relative expression of each transcript was calculated using the 2^–Δ^ ^Δ^ ^*Ct*^ method. RT-PCR reactions were performed for each sample in triplicate. For the set of reference samples, we used mRNA extracted from the patient, parents, and two additional healthy individuals (Bond et al., 2005). Data are presented in Figure 4 as mean ± SD (Bond et al., 2005).

### Ethics and Consent

Written informed consent was obtained from the patient’s parents for the publication of the case details and accompanying images. All research was conducted according to the Declaration of Helsinki, and the research protocol was registered with the following number #253. This study received approval from the Ethics Committee of Fundación Valle del Lili (Act 11/2016).

## Results

### Molecular Diagnosis

Serum and urine samples were tested for chikungunya virus, dengue virus, and ZIKV. The RT-quantitative PCR assays for dengue and chikungunya viruses were negative for whole samples and positive for ZIKV in the amniotic liquid of the patient but negative in the urine and serum samples from the fetus and mother. Serology tests of the serum and urine using anti-dengue virus IgM, anti-dengue-virus IgG, anti-chikungunya-virus IgM and IgG, and anti-ZIKV displayed negative results in the enzyme-linked immunosorbent assays (ELISAs). The samples also tested negative for TORCH (toxoplasmosis, HIV, rubella, syphilis, cytomegalovirus, hepatitis, parvovirus B19, and herpes simplex 1 and 2), as is described previously in the literature (Candelo et al., 2019).

### Karyotype and a-CGH

Fetal karyotyping displayed regular 46, XY (male) profiles. No losses and/or gains of genomic material were detected in the fetal DNA sample, as is described previously in the literature (Candelo et al., 2019).

### Whole-Exome Sequencing

**FIGURE 3:**
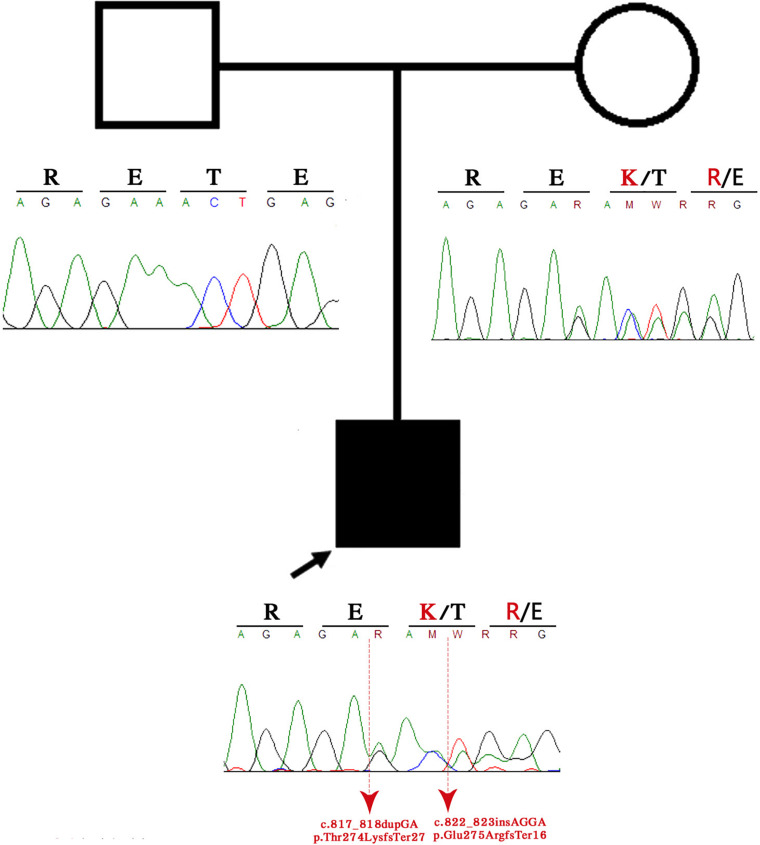
Family pedigree. The black arrow indicates the proband, and the sanger sequence represents the deleted region.

WES detected two heterozygous variants in the *CDK5RAP2* gene at positions p.Thr274LysfsTer27 and p.Glu275ArgfsTer16, respectively, when compared with the human reference template (GRCh38) or mutation databases (LOVD and HGMD). These variants result in a stop codon causing the early termination of the protein. Bioinformatic analysis was performed using the University of California Santa Cruz Genome Browser, which showed that the region was highly conserved between species and was located near to a regulatory element, such as H3K27Ac, and between a promoter and enhancer region. According to the AmiGO gene ontology browser and Kyoto Encyclopedia of Genes and Genomes, this gene has multiple functions, such as G2/M transition of the mitotic cell cycle, establishment of mitotic spindle orientation, microtubule cytoskeleton organization, maintaining centrosome function, spindle pole assembly and orientation, microtubule bundle formation, and protein binding (Fong et al., 2008; Figure 3).

### *In silico* Analysis

We identified two novel heterozygous nonsense mutations, both located in exon 8 under the affection of CZS, which produced a premature stop codon resulting in the truncation of CDK5RAP2 protein. There has been no pervious reporting of these variants in the literature. A functional prediction of the variants using gene prediction software classified them as harmful variants (disease-causing). At the moment, they are classified as “probably pathogenic” according to the American College of Medical Genetics and Genomics guidelines (see Table 2).

### Tissue Expression of CDK5RAP2

**FIGURE 4:**
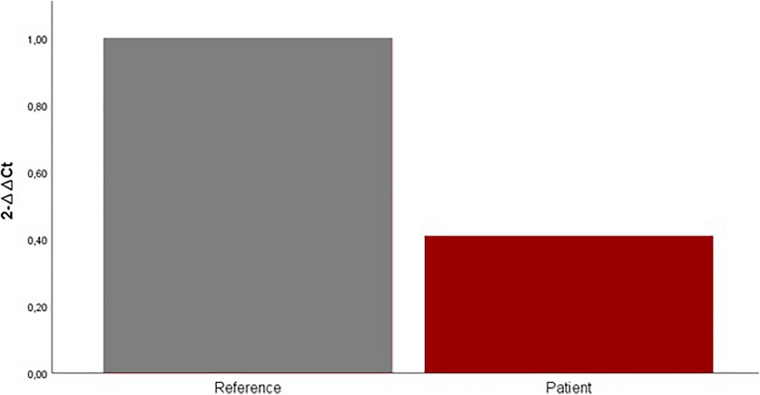
Expression levels of CDK5RAP2 measured by qRT-PCR in the patient (red column) and set of reference samples (gray column). Each GAPDH-normalized mRNA level was further normalized against the corresponding mRNA level. Data are shown as fold change relative to the set of reference samples (defined as 1.0). Data are represented as mean ± SD.

Quantitative gene expression analysis using real-time PCR showed that mRNA levels of CDK5RAP2 were downregulated in the patient mouth swab sample. Our results suggest an approximately 0.6-fold decrease of CDK5RAP2 expression in the patient mouth swab sample compared with the control mouth swab sample (Figure 4).

## Discussion

The term CZS was created to characterize the range of congenital abnormalities associated with ZIKV infection, including microcephaly to ocular injury and hearing loss (Walker et al., 2019). At the beginning of the ZKV outbreak, two fetuses from pregnant women from the state of Paraiba, Brazil were diagnosed with microcephaly and were thought to belong to the microcephaly cluster because they both suffered from symptoms related to ZIKV infection. In previous cases with central nervous system (CNS) abnormalities, brain atrophy, with coarse calcifications involving the white matter of the frontal lobes, including the caudate, lentostriatal vessels, and cerebellum, was mainly observed (Walker et al., 2019). The cerebral hemispheres were severely asymmetric, with marked unilateral ventriculomegaly, displacement of the midline, thinning of the parenchyma on the dilated side, failed visualization of the corpus callosum, and almost complete disappearance or failure to develop the thalami (Walker et al., 2019). The pons and brainstem were thin and continuous with a heterogenous small mass at the basal ganglia (Walker et al., 2019). The anomalies were limited to the brain in most cases. These brain abnormalities included cerebral calcifications, microcephaly, Blake’s pouch cyst, agenesis of the vermis, cerebral atrophy, mega cisterna magna, and ventriculomegaly (Walker et al., 2019). Maternal ZIKV infection is estimated to result in 5–13% of birth defects cases, with higher incidence when infection occurs earlier in pregnancy and microcephaly resulting in approximately 6.33% (interquartile range, 4–5.5%) of congenital ZIKV infections (Brasil et al., 2016; Honein et al., 2017; Shapiro-Mendoza et al., 2017; Hoen et al., 2018; Sanz Cortes et al., 2018; Shiu et al., 2018; Walker et al., 2018). The spectrum of anomalies associated with ZIKV is broad, and there are potential postnatal long-term neurocognitive deficits due to maternal–fetal exposure, which can lead to the loss of neural stem cells (neurogenic arrest) (Adams Waldorf et al., 2018).

Recent studies have suggested that placental injury and infarctions might affect fetal oxygenation (Hirsch et al., 2018). Furthermore, type I interferon triggered fetal death and altered placental development in mouse models of ZIKV infection (Yockey et al., 2018). Although the molecular mechanisms of ZIKV microcephaly remain unelucidated, it is understood that the flavivirus facilitates viral replication and spreading by dysregulating and bypassing the innate immune response to replicate in the unchecked host cells (Keller et al., 2006). Then, the virus kidnaps and modifies the genomic RNA in the colonized cells to mimic endogenous host cell processes, allowing the viral replication to remain undetected (Daffis et al., 2010). The synergic effect of ZIKV protein products interferes with the innate immune response by binding to the downstream interferon pathway (Donald et al., 2016) and disrupting Janus kinase signaling, which is fundamental for the expression of any antiviral factor (Grant et al., 2016). ZIKV directly infects and spreads in the neural stem cells in the fetus and produces neurogenic arrest by possible P53 activation and inhibition of the mTOR pathways, which promote a switch from glycolysis to oxidative phosphorylation and might consequently produce immature differentiation and apoptosis of neural precursor cells (NPCs) by targeting cell proliferation and generating cell cycle arrest, causing microcephaly and cortical thinning (Li et al., 2016).

Global gene analysis expression studies have demonstrated the upregulation and downregulation of multiple genes implicated in the immune response, apoptosis, and microcephaly (Li et al., 2016). There were few ZIKV-infected cells in the midbrain and the cortical plate, where post-mitotic neurons are based, ZIKV leads to cortex thinning without lamination disturbances, and in the mouse model, there were significantly fewer mitotic cells in the ventricular zone, accompanied by more centrosomes at the ventricular surface that were facing away from the nuclei (Li et al., 2016). Furthermore, the ZIKV-infected brain showed a lower number of NPCs in the M phase. In an experimental mouse model infected with ZIKV, global transcriptome analyses (RNA-sequencing) identified genes related to cytokine production and cytokine response; several genes related to cell proliferation, differentiation, and migration were downregulated; and most of the microcephaly-related genes were downregulated (*ASPM*, *CASC5*, *CENPF*, *MCPH1*, *RBBP8*, *STIL*, and *TBR2*). These genes were shown to share putative homologs that were co-expressed with CDK5RAP2, and molecular interaction occurred between *ASPM* and *CDK5RAP2* (Li et al., 2016). The gene disrupted in our present case indicates that *CDK5RAP2* might play a disruptive role in the arrest of ZIKV-infected NPCs. The products of many of these genes are enriched in NPCs and affect the mitotic cell behaviors centered around the centrosome and DNA repair. For example, a mutation in this compound heterozygous variant produces a stop codon mutation and decreased protein expression, which is translated as a decreased or loss of functional protein associated with a reduced number of NPCs due to asymmetric division (Merfeld et al., 2017). Another study proved that cerebral organoids created from human cells with disrupted CDK5RAP2 were smaller, contained fewer progenitors, and disrupted cell polarity (Lancaster et al., 2013). Research has also demonstrated that centrosome activity is more fundamental for the development of brain tissue than for any other tissue by associating *CDK5RAP2* gene disruption with microcephaly and ZIKV congenital infection (Merfeld et al., 2017).

The *MAPRE1* gene (also known as EB1) codes a protein that is located in the microtubules, especially at the growing ends, in interphase cells. The CDK5RAP2–MAPRE1 complex induces microtubule bundling and assembling, suggesting that this complex regulates microtubule dynamics. The mutations p.T274KTer27 and p.E275RTer16 produce a truncated protein with loss of the protein–protein interactions in the CDK5RAP2–MAPRE1 complex. This loss affects the self-assembling dynamics of the microtubules, resulting in the dysregulation of neuron production and brain size (Fong et al., 2009). CDK5RAP2 has a domain that is highly homologous to centrosomin motif 2, essential for pericentric interaction, which is fundamental for chromosomal and Golgi localization of the CDK5RAP2 protein. The resultant protein of the gene that contained the patient mutation lacks the centrosomin 2 domain, which suggests a loss in its localization to centrosomes and the Golgi complex as well as dysregulation of the cellular cycle, related to disturbances in microtubule dynamics (Wang et al., 2010). Model pregnant mice with ZIKV infection have been reported to show apoptosis, cell cycle arrest, and inhibition of NPC differentiation, generating the phenotype observed in our case (Figure 2), cortical thinning, and microcephaly. The disruption of CDK5RAP2 and the genes previously mentioned and the putative homologous co-expression of these genes might lead to microcephaly, with a lower NPC content and a disruption in cell polarity creating the phenotype observed in our patient (Merfeld et al., 2017).

Patients with autosomal-recessive primary microcephaly are widely known as having an isolated brain disorder without other system involvement. The present piece of evidence demonstrates the overlapping of the two syndromes on clinical manifestations (Tables 1,2). The proband exhibits the majority of congenital ZIKV signs and symptoms with some of the CDK5RAP2 characteristics and overlapping of both conditions (Table 1), which suggest and confirm assumptions that have been postulated by previous authors (Tan et al., 2014; Li et al., 2016), where it is proposed that ZIKV might not just disrupt the neural stem pool, but it could also interfere with gene expression and metabolic pathways in the same cells, which are crucial for neural development (Tan et al., 2014; Li et al., 2016). For that reason, we hypothesized that CDK5RAP2 variants might interact as a second hit effect in the existence of congenital ZIKV infection as in the present case their father is a carrier of p.Thr274LysfsTer27 and p.Glu275ArgfsTer16 without any specific phenotype. However, the proband has an overlapping phenotype of congenital ZIKV syndrome and CDK5RAP2 microcephaly disorder (Table 1 and Figures 3, 4). Furthermore, in the following studies, around 11 genes related to microcephaly have been demonstrated to be downregulated in the tissue from ZIKV-associated microcephaly, including the gene (*CDK5RAP2*) found in this case, which is also related and required for the maintenance of the germ cell pool during embryonic development and is linked with molecular evolutionary pathways for regulating the brain size (Evans et al., 2006; Zaqout et al., 2017). This indicates that the variable expressivity of the phenotype in patients with congenital ZIKV syndrome might be directly associated with the type and mechanism of gene disruption and the time of the viral exposure, which could generate the downregulation of these genes and it will be seen in patients as an absence of nucleotide variants compare with the human reference template in WES as was seen in the previous study (Candelo et al., 2019). In addition, severe affection might generate the genetic profile seen in this patient (Table 1 and Figure 3). Further investigation must focus on using genome-wide association data to confirm this association.

**TABLE 1:** Clinical manifestation overlap.

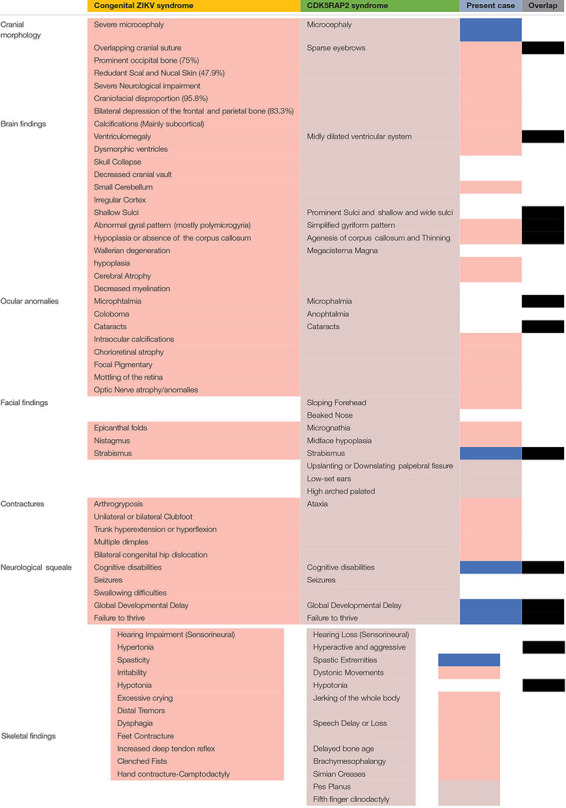

*Each column represents each clinical condition according to the literature. The clinical manifestation of congenital ZIKV syndrome is represented in yellow, and the CDK5RAP2 syndrome is represented in green. The present case column exhibits the symptoms and signs in the respective color that represents each syndrome, and blue shows the combination of a clinical manifestation shared from both conditions. The last column represents the overlap of clinical manifestation between the two conditions.*

**TABLE 2:** In silico analysis of the CDK5RAP2 variants.

**Variant**	**Protein effect**	**ClinVar**	**DANN**	**EIGEN**	**MutPred**	**MutationTaster**	**SIFT**	**PolyPhen-2**
c.1156C > T	p.Thr274LysfsTer27	NR	0.9987	Pathogenic	Pathogenic	Disease causing	Damaging	Probably damaging
c.280C > T	p.Glu275ArgfsTer16	NR	0.9993	Pathogenic	Pathogenic	Disease causing	Damaging	Probably damaging

In summary, our study describes a case of vertical ZIKV transmission associated with a congenital ZIKV syndrome and linked with a neurological syndrome, with microbiological, molecular, and genetic assessments, where two cis-novel variants in CDK5RAP2 were found. The *CDK5RAP2* gene encodes a pericentriolar protein that mediates microtubule nucleation and centriole attachment. A diallelic mutation on this gene has been previously associated with primary microcephaly.

## Data Availability Statement

The original contributions presented in the study are included in the article/supplementary material, further inquiries can be directed to the corresponding author/s.

## Ethics Statement

This study was approved by the Ethics Committee of Fundación Valle del Lili.

## Author Contributions

All authors made a substantial contribution to the work reported, whether that is in the conception, study design, execution, acquisition of data, analysis and interpretation, or in all these areas. They took part in drafting, revising, or critically reviewing the article and gave final approval of the version to be published. They have agreed on the journal to which the article has been submitted and agreed to be accountable for all aspects of the work.

## Conflict of Interest

The authors declare that the research was conducted in the absence of any commercial or financial relationships that could be construed as a potential conflict of interest.
